# Towards the development of novel *Trypanosoma brucei *RNA editing ligase 1 inhibitors

**DOI:** 10.1186/1471-2210-11-9

**Published:** 2011-08-30

**Authors:** Jacob D Durrant, J Andrew McCammon

**Affiliations:** 1 Department of Chemistry & Biochemistry, University of California San Diego, La Jolla, California 92093-0365, USA; 2 Department of Chemistry & Biochemistry and Department of Pharmacology and NSF Center for Theoretical Biological Physics, University of California San Diego, La Jolla, California 92093-0365, USA; 3 Howard Hughes Medical Institute, University of California San Diego, La Jolla, CA 92093-0365, USA

## Abstract

**Background:**

*Trypanosoma brucei *(*T. brucei*) is an infectious agent for which drug development has been largely neglected. We here use a recently developed computer program called AutoGrow to add interacting molecular fragments to **S5**, a known inhibitor of the validated *T. brucei *drug target RNA editing ligase 1, in order to improve its predicted binding affinity.

**Results:**

The proposed binding modes of the resulting compounds mimic that of ATP, the native substrate, and provide insights into novel protein-ligand interactions that may be exploited in future drug-discovery projects.

**Conclusions:**

We are hopeful that these new predicted inhibitors will aid medicinal chemists in developing novel therapeutics to fight human African trypanosomiasis.

## Background

*Trypanosoma brucei *(*T. brucei*) is an infectious agent for which drug development has been largely neglected [1]. *T. brucei *is endemic to Africa, where two subspecies fatal to humans exist [2]. Both subspecies can infect the central nervous system, where they cause the neurologic problems and general debilitation referred to as African sleeping sickness [3,4]. As current treatments are either expensive, toxic, or ineffective, new drugs are urgently needed. One potential novel *T. brucei *drug target is RNA editing ligase 1 (*Tb*REL1), a critical component of a unique mitochondrial RNA-editing complex called the editosome [5]. *Tb*REL1 is essential for *T. brucei *survival and has no close human homologues, making it an excellent drug target.

**Figure 1:**
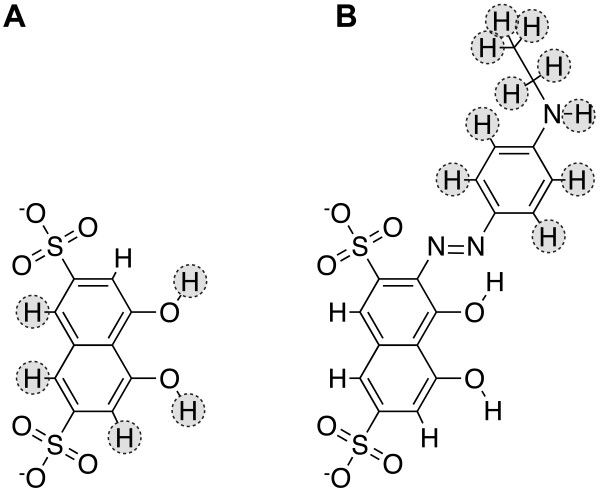
**The initial scaffolds used in AutoGrow runs**. Scaffold linker hydrogen atoms are highlighted in grey. a) 4,5-dihydroxynaphthalene-2,7-disulfonate, the initial scaffold used to generate the novel *Tb*REL1 inhibitors listed in Table 1. b) **S5**, the initial scaffold used to generate the novel *Tb*REL1 inhibitors listed in Tables 3 and S2 (Additional file 1).

Recently, Amaro et al. used a computational flexible-receptor strategy called the relaxed complex scheme to identify micromolar inhibitors of *Tb*REL1 [6]. One of these inhibitors, **S5 **(Figure 1b), had an approximate IC_50 _of 1 μM. Analysis suggested that some elements of **S5**-*Tb*REL1 binding might mimic ATP binding. Despite some similarities, however, **S5 **is not predicted to participate in many of the interactions that mediate ATP binding.

Motivated by the initial discovery of the **S5 **inhibitor and the desire to increase potency, we here use a drug-design program called AutoGrow 1.0 [7] to add interacting moieties to **S5 **in order to improve its predicted binding affinity.

## Results/Discussion

In the current work, we used the computer program AutoGrow 1.0 [7] to generate novel inhibitors of *Trypanosoma brucei *(*T. brucei*) RNA editing ligase 1 (*Tb*REL1) by adding interacting molecular fragments to **S5 **(Figure 1b), a recently discovered, experimentally verified *Tb*REL1 inhibitor [6].

**Figure 2:**
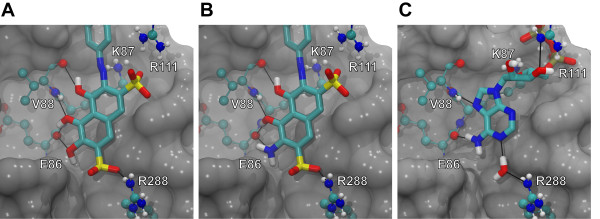
**The core of the two ligands listed in Table 2, as well as ATP, shown in detail**. The ligand poses of the novel compounds correspond to those of the lowest-energy AutoDock clusters; the ATP pose shown is crystallographic. A portion of the protein has been cut away to allow visualization of interactions deep in the *Tb*REL1 binding pocket. Selected hydrogen bonds are represented by black lines. Only polar hydrogen atoms are displayed.

Docking studies have suggested that some elements of **S5 **binding to *Tb*REL1 might mimic ATP binding (Figure 2c). Deep within the active site, **S5 **is predicted to form a hydrogen bond with the E86 backbone and to participate in π-π interactions with the F209 aromatic side chain, similar to the ATP adenine moiety. Additionally, one of the **S5 **sulfonate groups is predicted to replace a critical water molecule that participates in a hydrogen-bonding network between R288, D210, the backbone carbonyl oxygen atom of F209, Y58, and the N1 atom of the ATP adenine ring. Two of the **S5 **naphthalene hydroxyl groups are predicted to lie nearly coincident with the adenine N7 of ATP; the oxygen atoms of these two groups are predicted to accept hydrogen bonds from the backbone amine of V88, just as the ATP N7 atom does. Finally, a second sulfonate group likely forms electrostatic interactions with R111 and K87, thus mimicking, in part, the ATP polyphosphate tail [6].

Despite these similarities, **S5 **does not interact with many of the *Tb*REL1 hydrogen-bond donors and acceptors that mediate ATP binding. For example, there are no predicted interactions between **S5 **and E159 or N92. While **S5 **may participate in π-cation interactions with R309 and R111 at the active-site periphery, it apparently forms no hydrogen bonds with K307 or K87. We hypothesize that interacting molecular fragments can be added to the **S5 **scaffold to increase potency by mimicking additional protein-ATP interactions.

### How effective is virtual screening at identifying *Tb*REL1 inhibitors?

AutoGrow 1.0 is an evolutionary algorithm that evaluates the "fitness" of generated compounds by docking those compounds into the target receptor using AutoDock [8] and comparing the predicted binding energies. The reliability of AutoGrow is thus tied to the reliability of AutoDock itself. Fortunately, AutoDock 4.0 has been used extensively to identify experimentally validated *Tb*REL1 inhibitors [6,9]. For example, using virtual screening with AutoDock, Amaro et al. recently identified fourteen predicted *Tb*REL1 inhibitors, five of which were ultimately validated by experiment [6]. Among the true positives, AutoDock was able to distinguish between strong and weak inhibitors. A follow-up study used AutoDock to find additional naphthalene-based *Tb*REL1 inhibitors. One of these inhibitors was even effective against the whole-cell organism [9].

In our experience, the utility of docking programs in general is highly system dependent; for some receptors, computer docking provides little enrichment, but for others, docking is remarkable in its ability to identify true binders. Fortunately, past work has demonstrated that the *Tb*REL1 system is among those that are highly amenable to computer docking with AutoDock 4.0.

To further confirm that AutoDock is well suited to *Tb*REL1 docking, we performed a positive-control docking of ATP, the native substrate, into a *Tb*REL1 crystal structure. AutoDock works by docking a given compound into the target receptor multiple times and subsequently clustering all dockings by RMSD. The centriod members of the lowest-energy and the most-populated cluster are both arguably candidates for the "best" binding pose. In the case of ATP docking, the most-populated and lowest-energy AutoDock clusters were one and the same; additionally, the centriod member of this same cluster had an ATP binding pose very similar to that of the crystallographic structure (Figure 2c).

Aside from accurately predicting the energy of binding, computer docking should, ideally, also correctly predict the ligand binding pose. Indeed, the optimization described in the current study presupposes a correct understanding of ligand binding. Fortunately, computational evidence supports the supposition that the experimentally validated naphthalene-based *Tb*REL1 inhibitors on which the current work is based do in fact bind the ATP-binding pocket rather than a distant, allosteric site. First, one recent study showed that redocking with the AutoDock 4.0 scoring function can capture the crystallographic pose of a characterized ligand to within 2.5 Å RMSD 81% of the time [10]; that AutoDock would place naphthalene-based inhibitors snugly in the adenine-binding pocket in a pose that is both plausible and reminiscent of the binding of the natural substrate (ATP) is therefore promising. Second, it is difficult explain the two successful applications of AutoDock to this system, efforts that have lead to validated inhibitors effective against the protein target and the whole-cell parasite [6,9], if the docking poses on which those efforts are based are not targeting the correct pocket.

### Optimizing Interactions with Protein Residues Deep within the *Tb*REL1 Active Site

To optimize interactions between the protein and the **S5 **naphthalene, predicted to bind deep within the ATP-binding site [6], we first removed the portion of **S5 **predicted to interact with protein residues at the active-site periphery. After pruning, only 4,5-dihydroxynaphthalene-2,7-disulfonate (the "core") remained (Figure 1a). Multiple AutoGrow runs using the core as the initial scaffold and the score of the most-populated AutoDock cluster as the fitness metric produced only two compounds predicted to bind better than the core itself (Table 1). The same small-molecule fragments that AutoGrow added to 4,5-dihydroxynaphthalene-2,7-disulfonate to form these two top predicted binders were subsequently added to **S5 **using Discovery Studio (Accelrys) (compounds *c *and *d *of Table 2).

**Table 1:** Ligand modifications to enhance interactions with protein residues deep in the binding pocket

ID	Compound	Energy (kcal/mol)
A		-10.78
B		-10.22

To try to improve binding, modifications were made to the **S5 **core (4,5-dihydroxynaphthalene-2,7-disulfonate), the portion of **S5 **predicted to bind deep within the *Tb*REL1 binding pocket. AutoGrow generated only two compounds with greater predicted binding affinities than the core itself (compounds *a *and *b*). The predicted binding energy associated with the lowest-energy AutoDock cluster (Energy) is listed in the second column.

**Table 2:** Druglike properties of predicted ligands

ID	Structure	Energy (kcal/mol)	Weight (daltons)	HBA	HBD	LogP
C		-12.87	481.03	11	4	2.59
D		-13.11	480.04	10	5	2.40

Two ligands generated by modifying the 4,5-dihydroxynaphthalene-2,7-disulfonate core of **S5**. Listed with each compound is the AutoDock-predicted binding energy of the lowest-energy AutoDock cluster (Energy), as well as chemical properties computed using ICM 3.7, including molecular weight (Weight), the number of hydrogen bond acceptors (HBA), the number of hydrogen bond donors (HBD), and the predicted LogP.

However, when these modified compounds were docked into the *Tb*REL1 active site, it was the pose associated with the lowest-energy cluster, not the most-populated cluster, that positioned the naphthalene deep within the ATP-binding pocket. When the predicted binding energy associated with the lowest-energy AutoDock cluster was considered, both compounds *c *and *d *had improved predicted binding affinities over **S5 **(-12.87 and -13.11 kcal/mol, respectively, vs. -12.18 ± 0.32 kcal/mol for **S5**).

Analysis of the lowest-energy poses of compounds *c *and *d *revealed a number of predicted protein-ligand interactions deep within the binding pocket (Figure 2). In generating these two compounds, AutoGrow consistently added a hydrogen-bond donor at the three position of the naphthalene ring. Careful inspection of both compounds docked into the *Tb*REL1 active site revealed that these hydrogen-bond donors were docked nearly coincident with the amino group of the ATP adenine and may interact with the E86 backbone carbonyl oxygen atom, just as ATP does (Figure 2c). Other protein-ligand interactions deep within the binding pocket, similar to those that characterize **S5 **and ATP binding, are also evident (Figure 2).

### Optimizing Interactions with Protein Residues at the Active-Site Periphery

AutoGrow was also used to add fragments to **S5 **that interact with the protein at the active-site periphery (Tables 3 and S2 in Additional file 1). In all runs, AutoGrow found compounds that bound with an improved predicted binding affinity over **S5 **(-12.18 ± 0.32 kcal/mol) and over the co-crystallized ATP substrate (-10.44 kcal/mol), as measured by the predicted binding energy associated with the most-populated AutoDock cluster. The best compound is predicted to have a binding energy of -17.30 kcal/mol.

**Table 3:** Ligand modifications to enhance interactions with protein residues at the active-site periphery

ID	Structure	Energy (kcal/mol)	Weight (daltons)	HBA	HBD	LogP
E		-17.30	824.05	20	5	0.97
F		-17.21	813.05	20	5	0.82
G		-16.60	661.03	16	4	1.71
H		-16.60	757.01	17	4	3.14

The four best-scoring, unique, error-free ligands were selected from each of five AutoGrow runs. The top four best-scoring ligands of these twenty compounds are shown. Additional compounds are given in the additional files (Additional file 1, Table S2). Listed with each compound is the AutoDock-predicted binding energy of the most-populated AutoDock cluster (Energy), as well as chemical properties computed using ICM 3.7, including molecular weight (Weight), the number of hydrogen bond acceptors (HBA), the number of hydrogen bond donors (HBD), and the predicted LogP.

**Figure 3:**
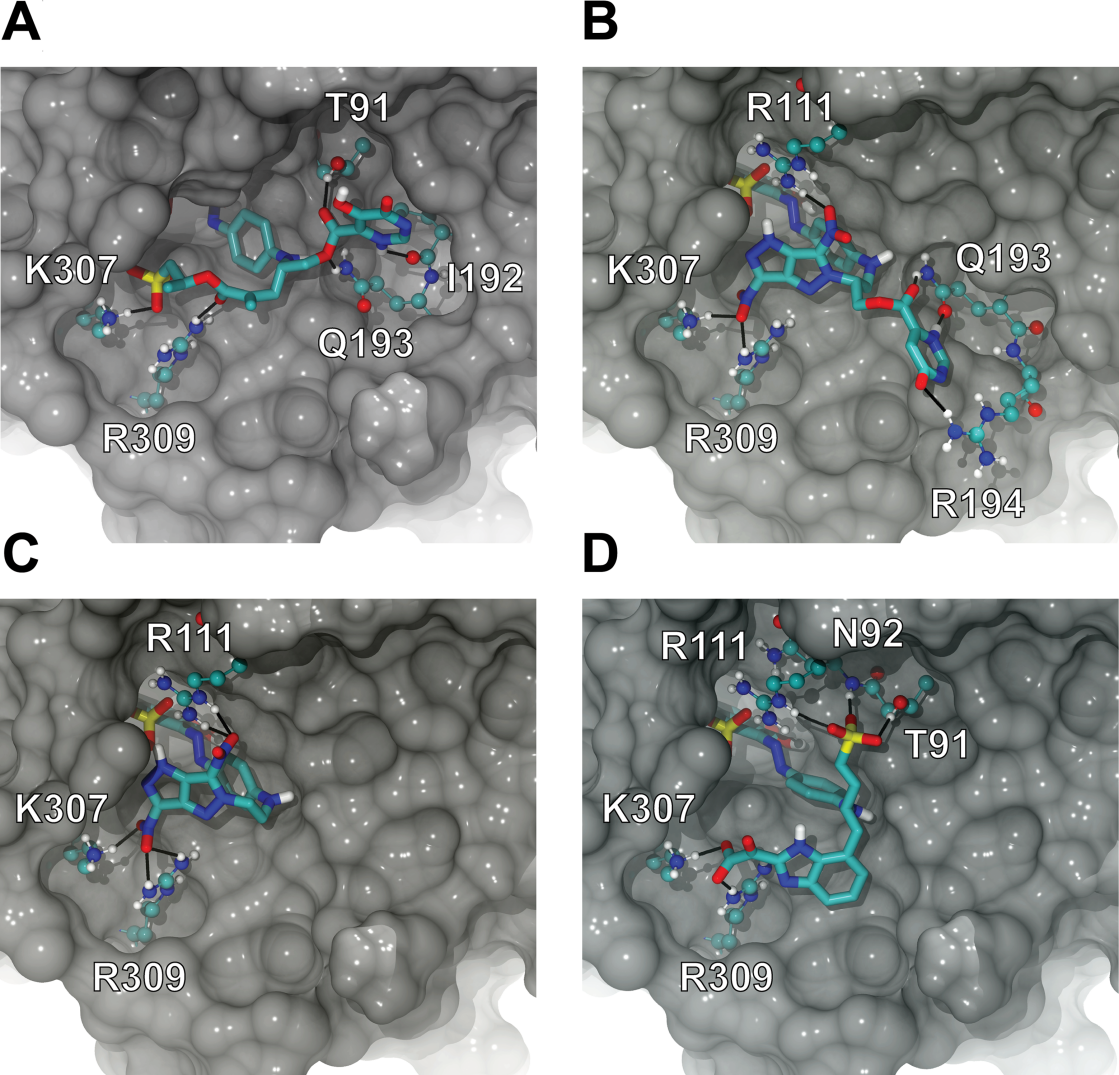
**The top four ligands (Table 3) docked into the *Tb*REL1 active site, shown in detail**. The ligand poses correspond to those of the most-populated AutoDock clusters. Potential hydrogen bonds at the active-site periphery are represented by black lines. Only polar hydrogen atoms are displayed.

AutoDock Tools (ADT 1.5.2) was used to analyze the top four predicted inhibitors (Table 3, Figure 3). Most of the predicted hydrogen-bond interactions at the active-site periphery involve side-chain amino or guanidine groups; K307, R309, R111, R194, and Q193 are all possible hydrogen bond donors. Side-chain oxygen atoms also participate in a few protein-ligand hydrogen bonds; T91 can donate a hydrogen bond via its hydroxyl group, and Q193 can accept a hydrogen bond via its side-chain carbonyl oxygen atom.

Among the top four ligands, interactions with the protein backbone were also observed. The backbone carbonyl oxygen atom of I192 can accept a hydrogen bond, and the backbone amine of N92 can donate. Of all these hydrogen-bond participants at the periphery, R309 and N92 are of particular interest. R309 is conserved across all members of the nucleotidyltransferase superfamily, and N92 is conserved across all type II RNA ligases [11]. As drug resistance often arises due to point mutations, it is fortunate that these critical binding residues are essential and therefore less likely to be subject to point mutations that confer resistance.

The top four compounds (Table 3, Figure 3) are predicted to interact with protein residues near the active-site periphery in ways similar to the ATP polyphosphate tail. X-ray crystallography shows that a non-bridging oxygen atom of the ATP alpha phosphate accepts a hydrogen bond from the K307 amino group. When docked, compound *e*, compounds *f *and *g*, and compound *h *position a sulfonate group, nitro groups, and a carboxylate group at the same location, respectively. Similar to ATP, all of these groups are predicted to interact with the K307 amine. This predictive result is particularly interesting, as several experimentally validated *Tb*REL1 inhibitors have recently been discovered that place sulfonate groups near K307 as well. One of these compounds is even effective against the whole-cell pathogen [9].

One of the non-bridging oxygen atoms of the ATP beta phosphate accepts a hydrogen bond from the R309 guanidine. When docked, compounds *e *and *h *place carbonyl and carboxylate oxygen atoms, respectively, near the same location. These groups are likewise predicted to accept hydrogen bonds from the R309 guanidine, mimicking the ATP beta phosphate. Finally, one of the non-bridging oxygen atoms of the ATP gamma phosphate accepts a hydrogen bond from the R111 guanidine. When docked, compounds *f *and *g *both position nitro groups coincident with the ATP gamma phosphate, and compound *h *positions a sulfonate group at that general location, all of which are likewise predicted to accept hydrogen bonds from the R111 guanidine.

The successful identification of predicted ligands that mimic ATP binding represents an interesting new form of convergent evolution. *Tb*REL1, over millions of years, slowly evolved to accommodate ATP binding. In contrast, in the current work we used an evolutionary algorithm, AutoGrow 1.0, to evolve novel ligands predicted to better accommodate the *Tb*REL1 receptor. In both cases, remarkably similar solutions emerged.

## Conclusions

We here used the computer program AutoGrow 1.0 [7] to develop predicted inhibitors of *Trypanosoma brucei *RNA editing ligase 1 (*Tb*REL1), an experimentally validated drug target. AutoGrow produced a number of potential inhibitors (Tables 2, 3, and S2) that are predicted to bind *Tb*REL1 in ways similar to ATP binding; the best novel compound had a predicted binding energy of -17.30 kcal/mol.

ICM 3.7 (MolSoft) was used to evaluate the twenty-two novel compounds listed in Tables 2, 3, and S2 (Additional file 1) for drug-like properties. The compounds do not satisfy Lipinski's Rule of Five [12]; the average molecular weight (679.68 daltons) and the average number of hydrogen bond acceptors (15.95) are both too high. However, in other respects these compounds are drug like, with only 4.36 hydrogen bond donors on average and an average predicted LogP value of 1.96. Aside from falling short of satisfying Lipinski's Rule of Five, these AutoGrow-generated compounds also include a number of unfavorable ADME-Tox functional groups, including sulfonate, aniline, phenol, acrylate, vinyl-ether, carboxylic-acid, imidazole, and aminal groups.

Despite these weaknesses, AutoGrow has suggested a number of predicted potent inhibitors that chemists could conceivably optimize. For example, we note that some of the interacting fragments that AutoGrow added to the initial scaffold may only slightly increase the predicted binding energy; removal of these fragments may reduce the molecular weight of the compounds without abolishing ligand binding. Additionally, many of the heteroatoms of these compounds are not predicted to participate in hydrogen bonds with the protein receptor. The number of hydrogen-bond acceptors could be reduced without sacrificing binding affinity by replacing these atoms with carbon atoms. In fact, the removal of buried but unsatisfied hydrogen bond acceptors may improve binding by decreasing the energy of desolvation. Finally, unfavorable ADME-Tox functional groups could be replaced with acceptable groups that maintain the same hydrogen bond, electrostatic, and van der Waals interactions.

As ideal HAT therapeutics must be sufficiently hydrophobic to cross gut, blood-brain, and parasitic-membrane barriers, the two sulfonate groups of the predicted inhibitors herein described are concerning. However, examples of approved sulfonated drugs that are orally available (acamprosate and metamizole) and capable of entering the central nervous system (acamprosate) do exist. Additionally, an experimentally validated, doubly sulfonated naphthalene-based inhibitor was recently shown to be effective against whole-cell *T. brucei *[9], suggesting sufficient hydrophobicity to at least cross the parasitic cell membrane. Nevertheless, modification of one or more of these sulfonate groups will likely improve ADME/tox properties. A number of sulfonate bioisosteres, including the sulfonamide, could be considered.

Clearly, AutoGrow should be used to supplement the medicinal chemist's creativity rather than to replace it. We are hopeful that these predicted inhibitors will aid medicinal chemists in developing novel therapeutics to fight human African trypanosomiasis.

## Methods

### Optimizing Interactions Deep within the Active Site: Small-Fragment Modifications

To improve protein-ligand interactions deep within the active site, we first removed the portions of the **S5 **ligand predicted to interact with the protein at the active-site periphery. After pruning, only 4,5-dihydroxynaphthalene-2,7-disulfonate, i.e. the "core," remained (Figure 1a). We next ran the program AutoGrow 1.0 [7] for one generation using the core as the initial scaffold. 647 AutoGrow-generated "mutants" were created by drawing upon the default AutoGrow small-fragment library. Of these, only two docked within the *Tb*REL1 binding site.

The same small-fragment modifications made to the core were made to **S5 **itself. After minor geometrical corrections, the modified **S5 **ligands were docked into *Tb*REL1 using AutoDock 4.0.1 [8]. Predicted binding energies are shown in Tables 1 and 2. AutoDock docking parameters were similar to the parameters published previously for the positive-control docking of ATP into a *Tb*REL1 crystal structure [6] (Additional file 1, Table S1, parameter set A).

### Optimizing Interactions at the Active-Site Periphery: Large-Fragment Modifications

In order to identify novel inhibitors that have increased protein-ligand interactions at the active-site periphery, AutoGrow 1.0 was run five times, each time with the entire **S5 **molecule as the initial scaffold. All hydrogen atoms except those of the core served as linkers for fragment addition (Figure 1b).

The first three of the five AutoGrow runs each ran for nine generations. For the first eight generations, mutants were created by drawing upon the default large-fragment library. One additional generation was executed using the default small-fragment library, allowing for more precise refinements. Each generation initially consisted of 50 ligands. For each generation after the first, ten primary individuals were taken from the previous generation, based on both the score of the most populated AutoDock cluster and successful active-site docking (i.e., docking into the deep, well-defined, adenine-binding *Tb*REL1 pocket). An additional twenty "children" and twenty "mutants" were created from these ten primary individuals, subject to the requirement that all compounds contain fewer than seventy atoms. The first generation initially contained only the scaffold and 49 "mutants," as no previous generation existed from which "parents" could be drawn for crossover production.

Ligands were again docked into *Tb*REL1 (PDB: 1XDN) [13] using AutoDock 4.0.1 [8]. In order to increase the speed of the calculation, the AutoDock parameters were relaxed somewhat (Additional file 1, Table S1, parameter set B). Specifically, the maximum number of energy evaluations was reduced from 12 × 10^6 ^to 7 × 10^6^, and the number of runs was reduced from 100 to 25.

### Optimizing Interactions at the Active-Site Periphery: Small-Fragment Modifications

The fourth of the five AutoGrow runs used to generate compounds with novel interactions at the active-site periphery ran for three generations with the same relaxed AutoGrow and AutoDock parameters described above (Additional file 1, Table S1, parameter set B). Rather than drawing upon the default large-fragment library, all mutants were generated via the addition of fragments from the default small-fragment library, allowing for refinement of the initial scaffold without the major chemical changes that accompany large-fragment addition.

### Optimizing Interactions at the Active-Site Periphery: Fragment Recombination

For the final of the five AutoGrow runs used to generate compounds with novel interactions at the active-site periphery, a new fragment database was prepared. The AutoGrow-generated moieties of the top four error-free, unique ligands from each of the first four AutoGrow runs were isolated by removing the initial scaffold and replacing scaffold attachment points with hydrogen atoms using the PRODRG server [14]. The isolated moieties were then rescored with the AutoDock 4.0 force field without redocking, thereby associating a score (predicted binding energy) with each posed fragment. A script was then used to identify all possible combinations of these fragments that were mutually geometrically compatible, such that no two moieties in the combination came within 2 Å of each other. These combinations were then ranked by the sum of the predicted binding energies of their constituent fragments. A new fragment library was created by taking the union of the top five scoring combinations, which contained a total of seven unique fragments that were both strongly binding and mutually sterically compatible. All polar fragment hydrogen atoms were retained for subsequent use in AutoDock, so that fragment linker hydrogen atoms included both the original linker hydrogen atom as well as any polar hydrogen atoms.

AutoGrow was then executed for eight generations using this new fragment library. Each generation initially contained 100 ligand models. For each generation after the first, fifty individuals were taken from the previous generation, based on both the score of the most-populated AutoDock cluster and successful active-site docking. An additional fifty "children" were created from these fifty initial individuals, without any restraints on the size of the evolving compounds. The first generation initially contained only the scaffold model and 99 "mutants" created by drawing upon the new fragment library. All mutants were generated by replacing with fragments only those scaffold linker hydrogen atoms that the seven fragments of the new library had previously replaced, or their chemical equivalents. Ligands were docked to *Tb*REL1 using the same AutoDock parameters described above.

The four best-scoring, unique ligands were selected from each of the five AutoGrow runs, yielding the twenty compounds listed in Tables 3 and S2 (Additional file 1). As a beta version of AutoGrow was used, in some rare cases the AutoGrow crossover operator incorrectly created compounds with two distinct, overlapping fragments connected to the same scaffold linker hydrogen atom. As these compounds are not possible in nature, they were discarded, and the next best ligand from the corresponding AutoGrow run was considered.

### Optimizing Interactions at the Active-Site Periphery: Rescoring

In the five AutoGrow runs executed to increase ligand-protein interactions at the active-site periphery, less than ideal AutoDock parameters were used in order to increase the speed of the calculation. For example, the AutoDock-predicted binding energy may not have fully converged because the maximum number of energy evaluations was set to only 7 × 10^6^. Additionally, as the number of runs was set to 25, the most-populated AutoDock cluster may not have been statistically significant. Consequently, the twenty compounds were redocked using a more rigorous AutoDock parameter set (Additional file 1, Table S1, parameter set A). Energies reported in Tables 3 and S2 (Additional file 1) were calculated using these rigorous AutoDock parameters.

## Authors' contributions

JDD designed and executed the study. He also drafted the manuscript. JAM likewise participated in the writing and editing of the text. All authors read and approved the manuscript.

## Supplementary Material

Additional file 1 **Supporting Information**. Table S1 describes the two sets of AutoDock parameters that were used in the current study. Table S2 is an expanded version of Table 3 that shows modified compounds with enhanced interactions at the active-site periphery. Click here for file

## References

[B1] Remme JH Blas E Chitsulo L Desjeux PM Engers HD Kanyok TP Kengeya Kayondo JF Kioy DW Kumaraswami V Lazdins JK Strategic emphases for tropical diseases research: a TDR perspective Trends in parasitology 2002 18 10 421 426 10.1016/S1471-4922(02)02387-5 12377584

[B2] Barrett MP Boykin DW Brun R Tidwell RR Human African trypanosomiasis: pharmacological re-engagement with a neglected disease BrJPharmacol 2007 152 8 1155 1171 10.1038/sj.bjp.0707354 PMC2441931 17618313

[B3] Barrett MP Burchmore RJ Stich A Lazzari JO Frasch AC Cazzulo JJ Krishna S The trypanosomiases Lancet 2003 362 9394 1469 1480 10.1016/S0140-6736(03)14694-6 14602444

[B4] Buguet A Bert J Tapie P Tabaraud F Doua F Lonsdorfer J Bogui P Dumas M Sleep-Wake Cycle in Human African Trypanosomiasis J Clin Neurophysiol 1993 10 190 196 10.1097/00004691-199304000-00006 8389383

[B5] Stuart K Allen TE Heidmann S Seiwert SD RNA editing in kinetoplastid protozoa MicrobiolMolBiolRev 1997 61 1 105 120 10.1128/mmbr.61.1.105-120.1997 PMC232603 9106367

[B6] Amaro RE Schnaufer A Interthal H Hol W Stuart KD McCammon JA Discovery of drug-like inhibitors of an essential RNA-editing ligase in Trypanosoma brucei Proceedings of the National Academy of Sciences 2008 105 45 17278 17283 10.1073/pnas.0805820105 PMC2577703 18981420

[B7] Durrant JD Amaro RE McCammon JA AutoGrow: A Novel Algorithm for Protein Inhibitor Design Chemical Biology & Drug Design 2009 73 2 168 178 10.1111/j.1747-0285.2008.00761.x 19207419 PMC2724963

[B8] Morris GM Goodsell DS Halliday RS Huey R Hart WE Belew RK Olson AJ Automated docking using a Lamarckian genetic algorithm and an empirical binding free energy function Journal of Computational Chemistry 1998 19 14 1639 1662 10.1002/(SICI)1096-987X(19981115)19:14<1639::AID-JCC10>3.0.CO;2-B

[B9] Durrant JD Hall L Swift RV Landon M Schnaufer A Amaro RE Novel Naphthalene-Based Inhibitors of Trypanosoma brucei RNA Editing Ligase 1 PLoS Negl Trop Dis 2010 4 8 e803 10.1371/journal.pntd.0000803 20808768 PMC2927429

[B10] Goodsell DS Huey R Morris GM Olson AJ A semiempirical free energy force field with charge-based desolvation Journal of Computational Chemistry 2007 28 6 1145 1152 10.1002/jcc.20634 17274016

[B11] Amaro RE Swift RV McCammon JA Functional and Structural Insights Revealed by Molecular Dynamics Simulations of an Essential RNA Editing Ligase in Trypanosoma brucei PLoS Negl TropDis 2007 1 2 e68 10.1371/journal.pntd.0000068 PMC2100368 18060084

[B12] Lipinski CA Lombardo F Dominy BW Feeney PJ Experimental and computational approaches to estimate solubility and permeability in drug discovery and development settings Adv Drug Deliv Rev 2001 46 1-3 3 26 10.1016/S0169-409X(00)00129-0 11259830

[B13] Deng J Schnaufer A Salavati R Stuart KD Hol WG High resolution crystal structure of a key editosome enzyme from Trypanosoma brucei: RNA editing ligase 1 JMolBiol 2004 343 3 601 613 10.1016/j.jmb.2004.08.041 15465048

[B14] Schuttelkopf AW van Aalten DM PRODRG: a tool for high-throughput crystallography of protein-ligand complexes Acta Crystallogr D Biol Crystallogr 2004 60 Pt 8 1355 1363 15272157 10.1107/S0907444904011679

