# Supplementary material for: Towards the development of novel Trypanosoma brucei RNA editing ligase 1 inhibitors
Source: BMC Pharmacol. 2011 Aug 30;11:9. doi: 10.1186/1471-2210-11-9 (PMC3196686; doi:10.1186/1471-2210-11-9)
Supplement: Additional file 1 — Supporting Information. Table S1 describes the two sets of AutoDock parameters that were used in the current study. Table S2 is an expanded version of Table 3 that shows modified compounds with enhanced interactions at the active-site periphery. [file 1471-2210-11-9-S1.DOC]

|  | A | B |
| --- | --- | --- |
| ga_pop_size | 150 | 150 |
| ga_num_evals | 1.2 x 10^7^ | 7 x 10^6^ |
| ga_run | 100 | 25 |
| rmstol | 2.0 Å | 2.0 Å |
| best-cluster  criteria | most-populated | most-populated |

**Table S1**. Two sets of AutoDock parameters were used in the current study. Parameter set A was less rigorous but allowed for faster ligand evolution. Parameter set B was more rigorous and time consuming. Default values were used for all AutoDock parameters not listed. For all runs, AutoGrid 4.0 was used to generate affinity grids centered on the active site. Each grid enclosed a volume of 32.25 Å x 27.0 Å x 29.25 Å with 0.375 Å spacing.

| ID | Structure | Energy  (kcal/mol) | Weight  (daltons) | HBA | HBD | LogP |
| --- | --- | --- | --- | --- | --- | --- |
| I | 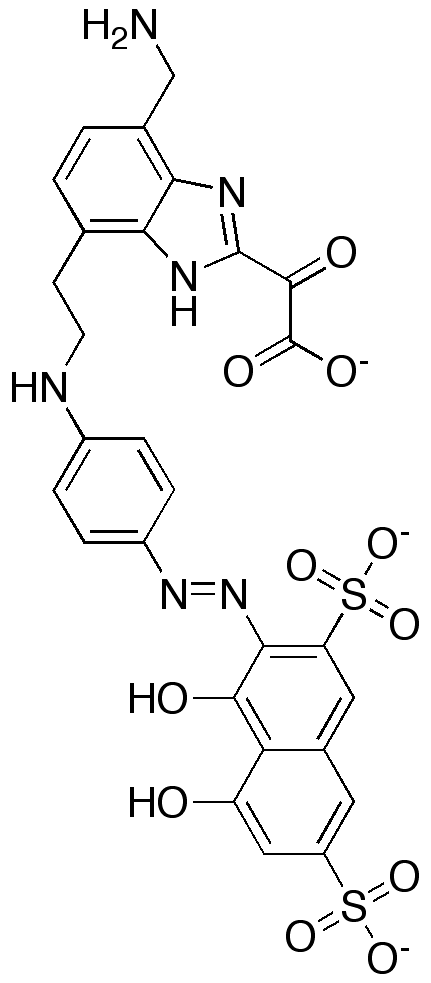 | -16.17 | 681.07 | 15 | 6 | 1.85 |
| J | 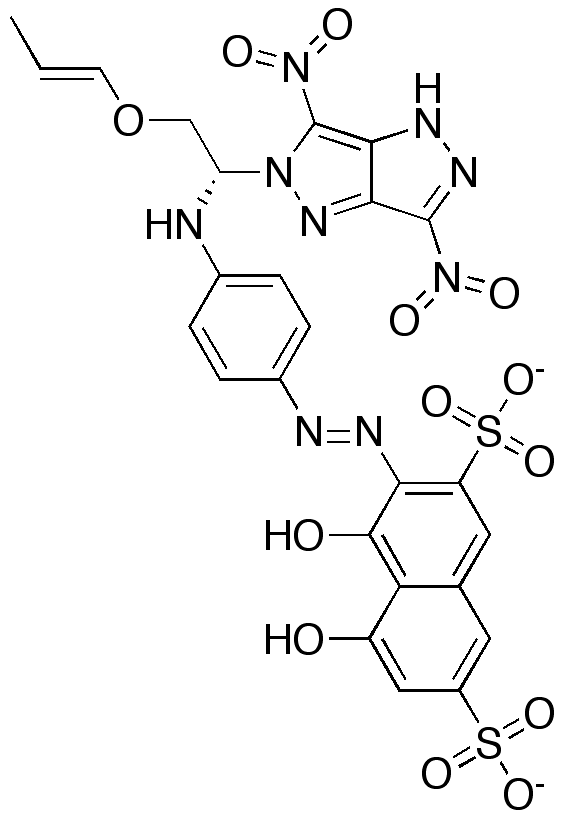 | -16.15 | 717.05 | 17 | 4 | 2.71 |
| K | 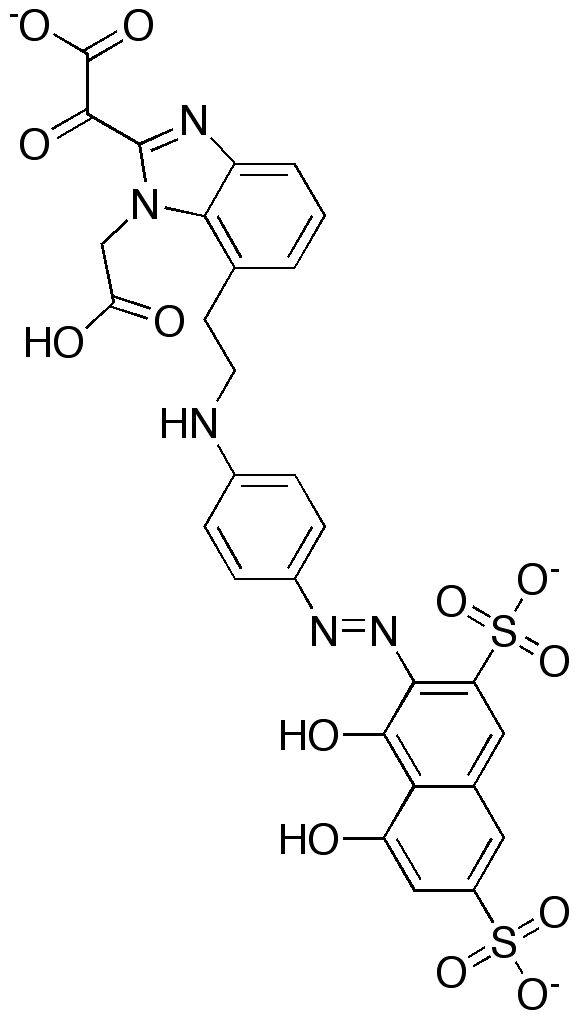 | -15.86­­­ | 710.05 | 16 | 4 | 2.62 |
| L | 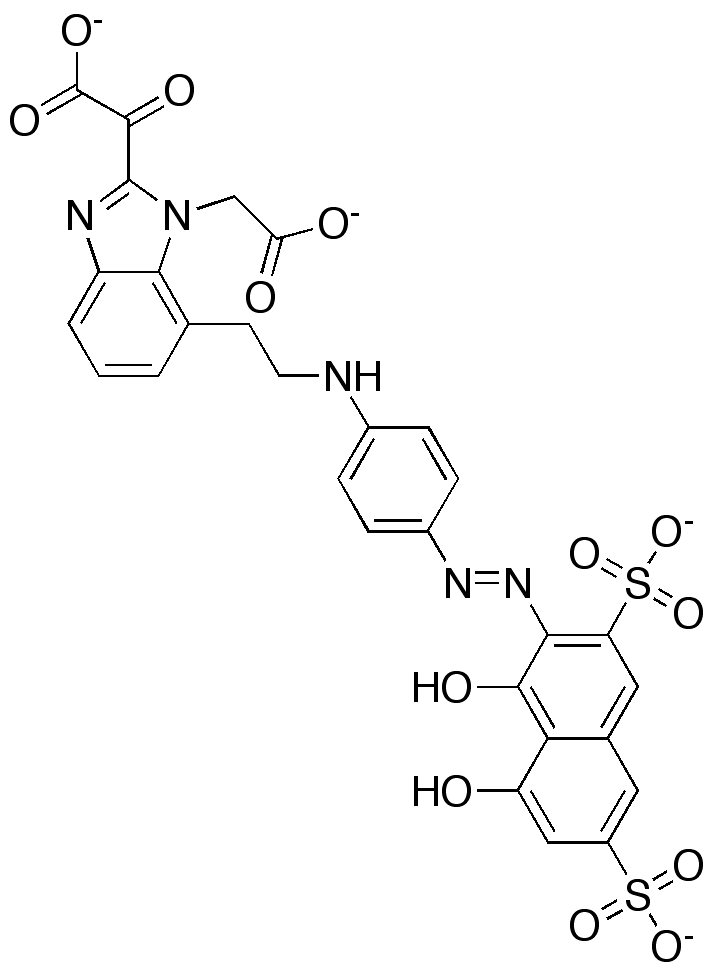 | -15.60 | 709.04 | 16 | 3 | 2.69 |
| M | 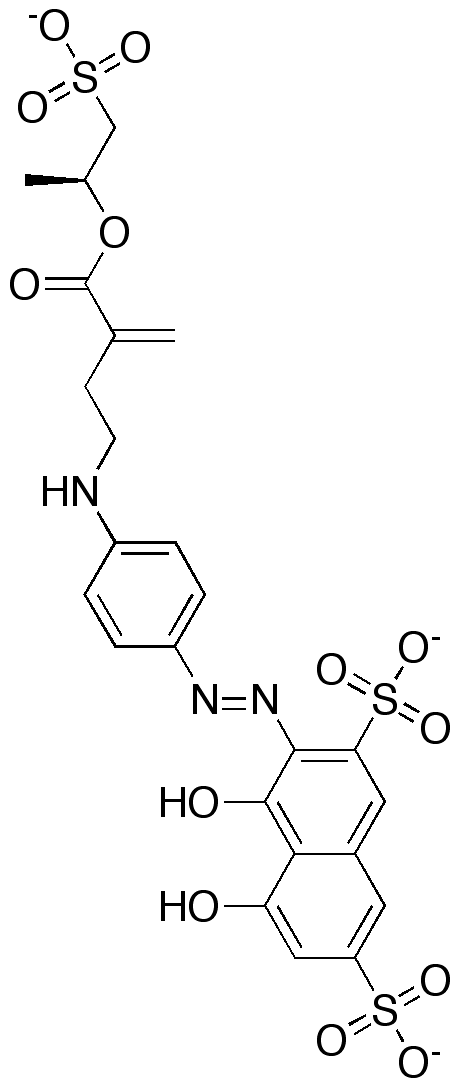 | -15.29 | 656.03 | 15 | 3 | 2.45 |
| N | 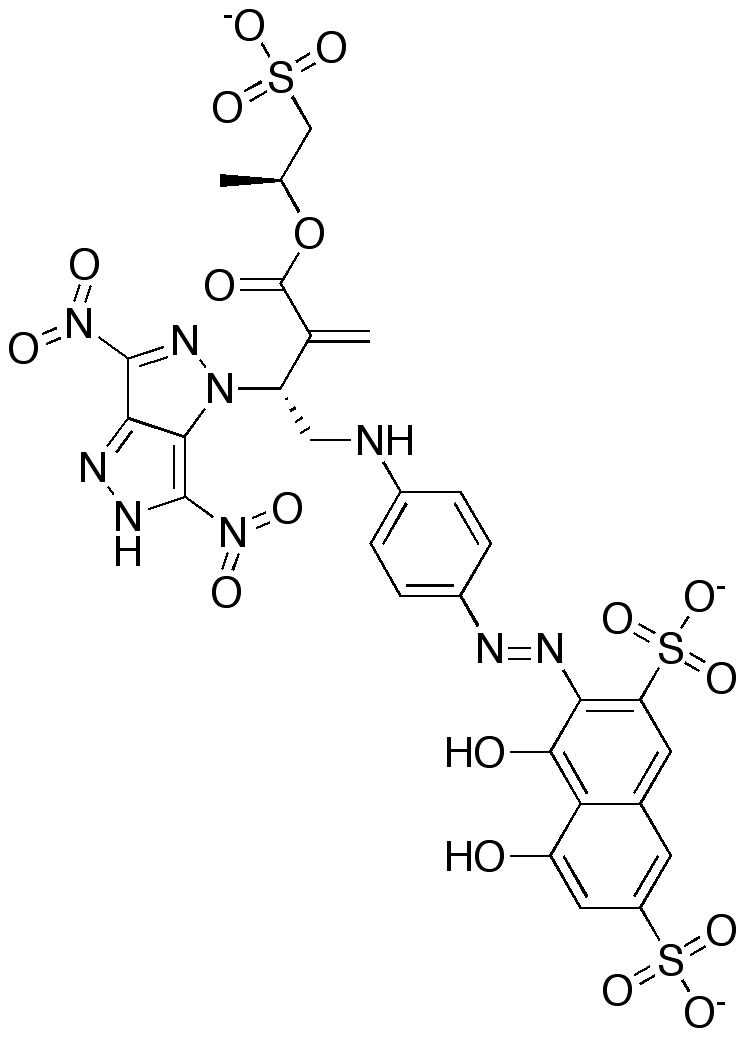 | -15.28 | 852.03 | 21 | 4 | 1.24 |
| O | 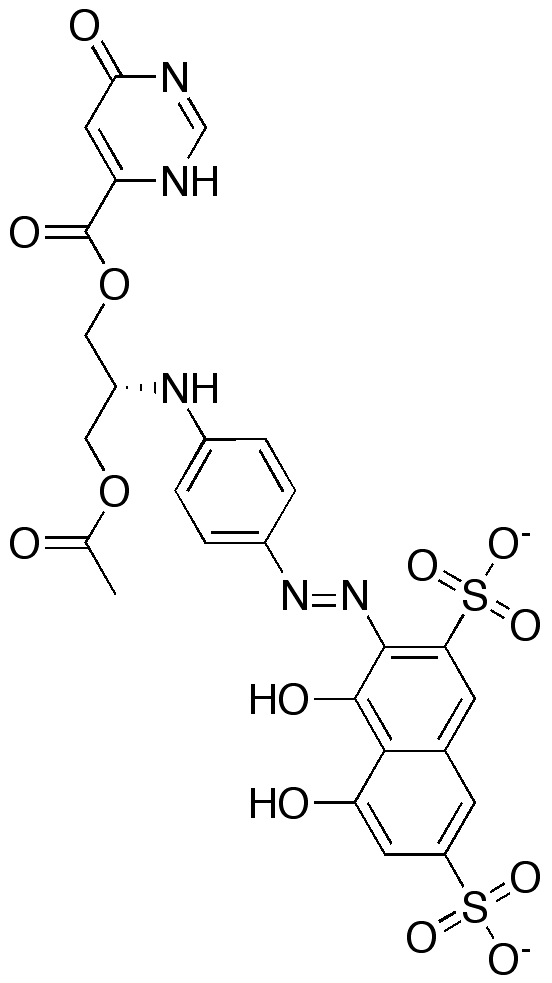 | -15.27 | 675.06 | 16 | 4 | 1.59 |
| P | 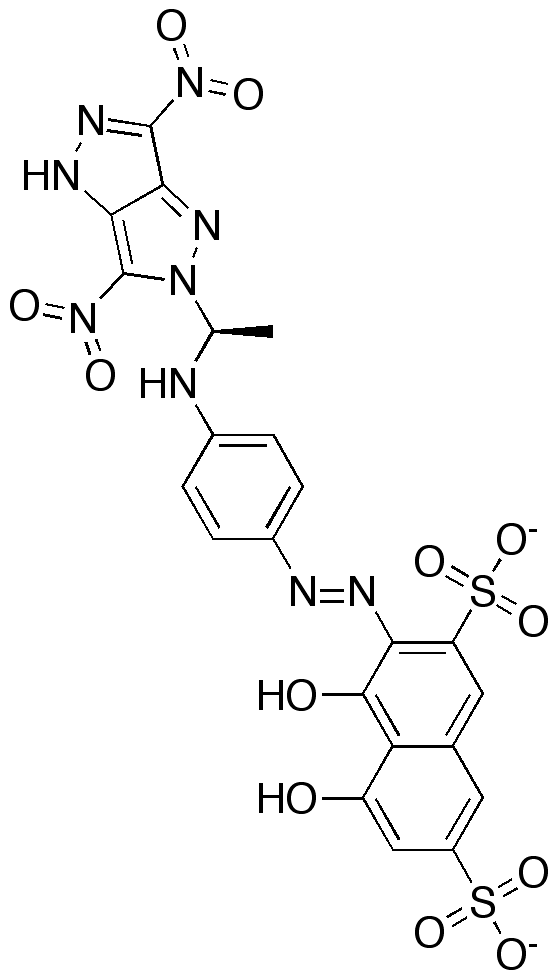 | -14.90 | 661.03 | 16 | 4 | 2.03 |
| Q | 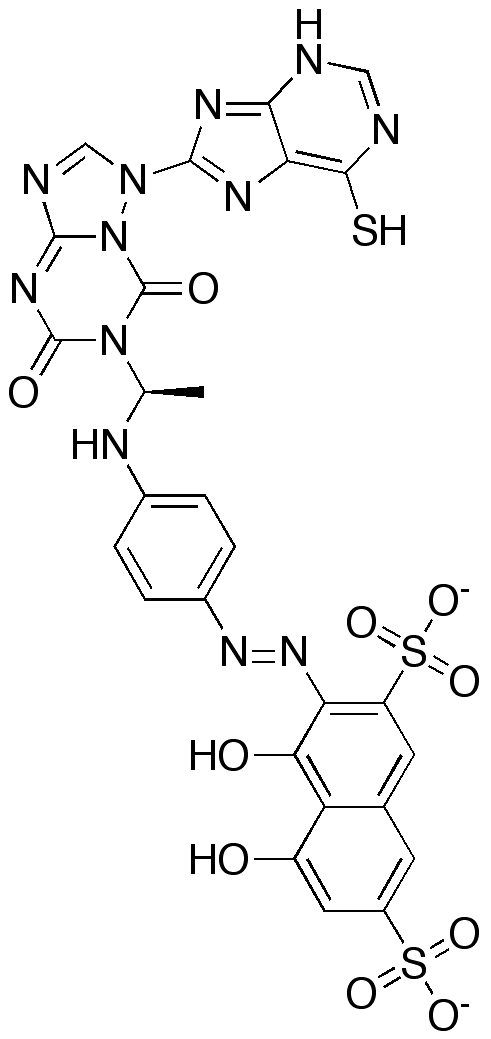 | -14.74 | 766.04 | 18 | 5 | -0.13 |
| R | 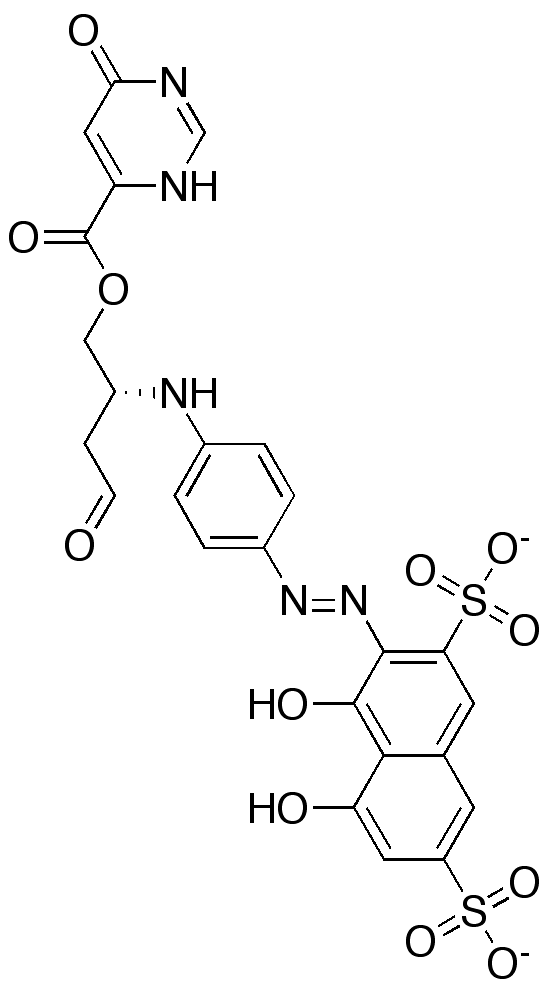 | -14.62 | 645.05 | 15 | 4 | 1.36 |
| S | 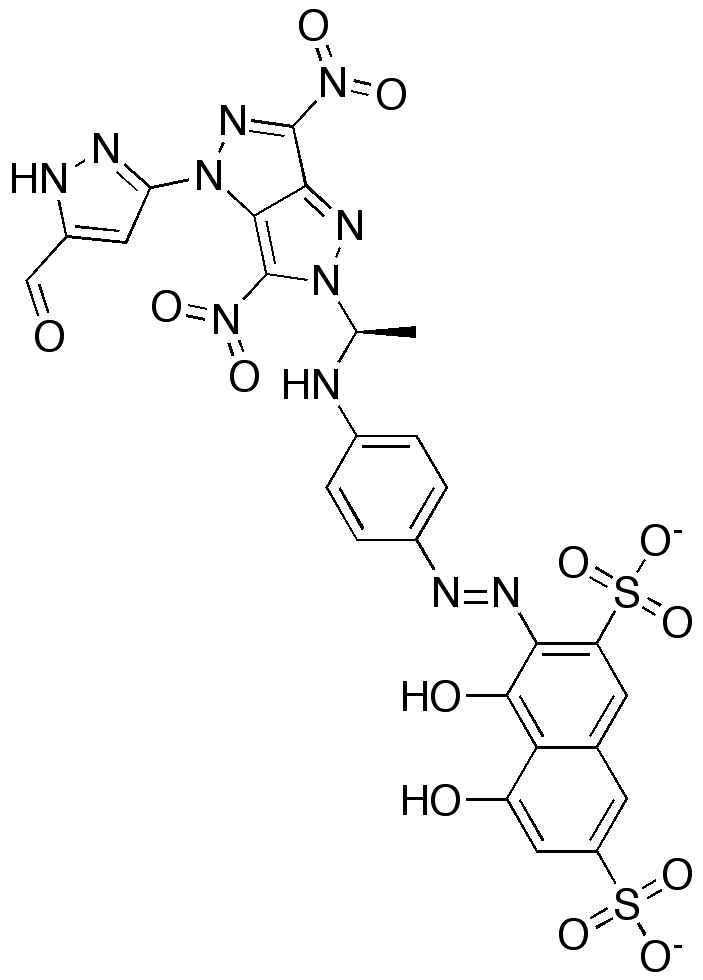 | -14.19 | 755.04 | 18 | 4 | 1.96 |
| T | 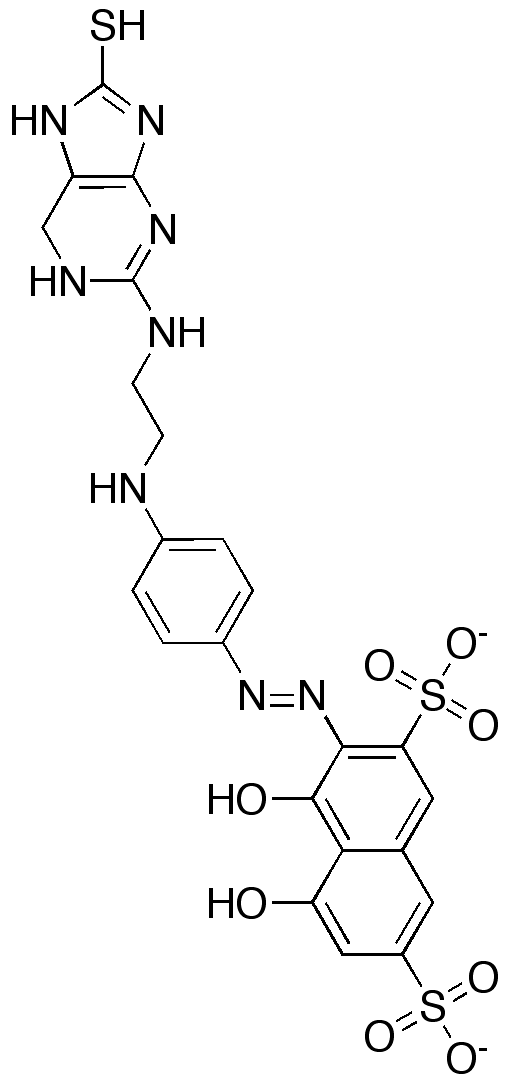 | -13.82 | 634.07 | 15 | 7 | 2.16 |
| U | 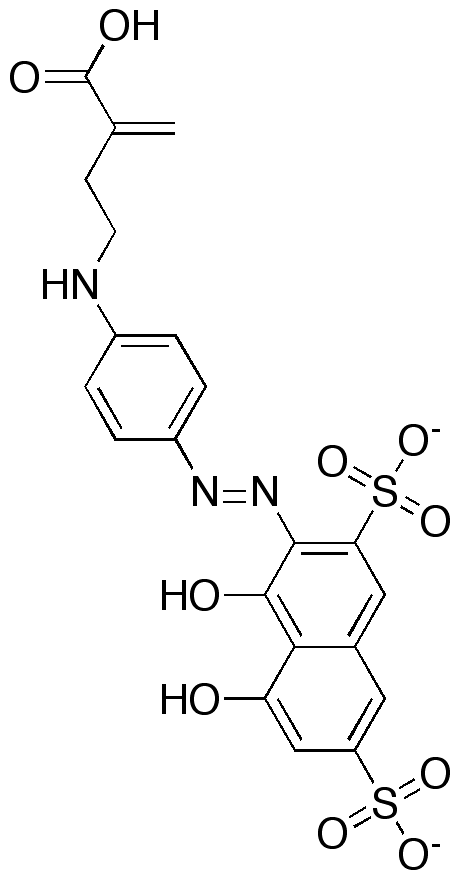 | -13.43 | 535.04 | 12 | 4 | 3.04 |
| V | 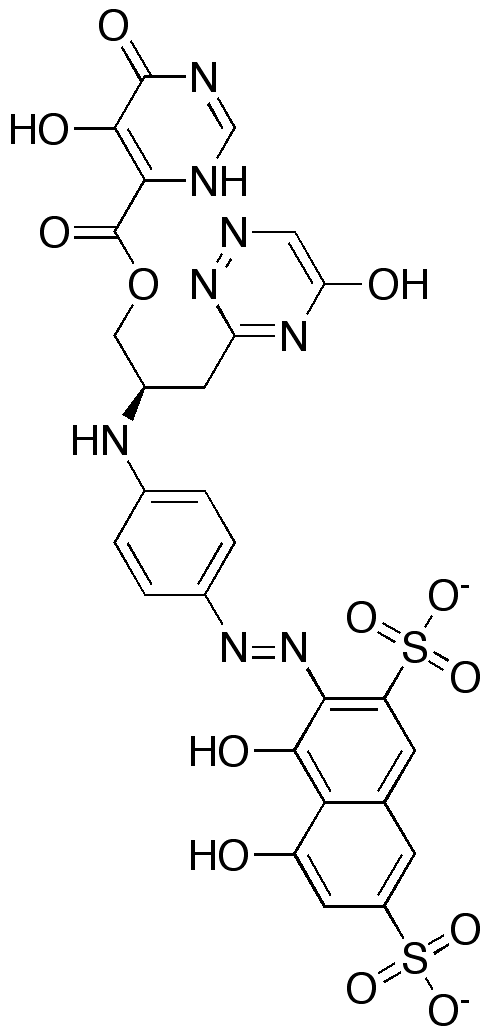 | -13.06 | 728.06 | 19 | 6 | 1.09 |
| W | 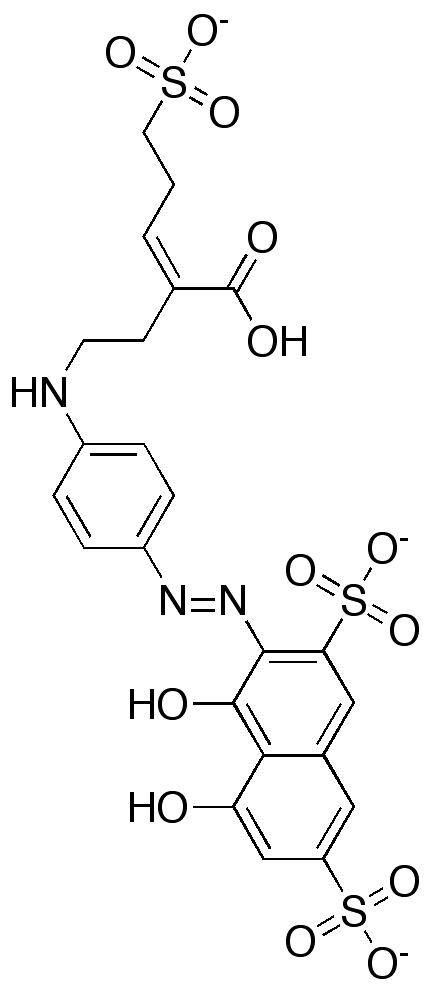 | -13.06 | 642.02 | 15 | 4 | 2.14 |
| X | 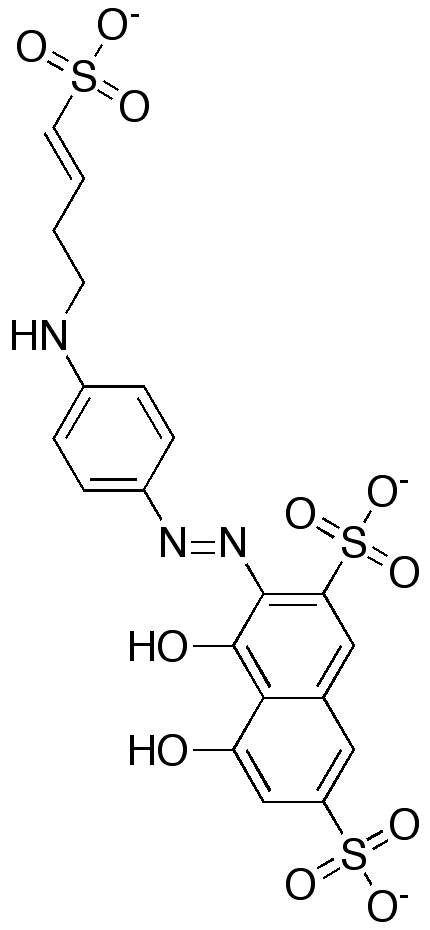 | -13.02 | 569.99 | 13 | 3 | 2.76 |

**Table S2.** *Ligand modifications to enhance interactions with protein residues at the active-site periphery*. The four best-scoring, unique, error-free ligands were selected from each of five AutoGrow runs. The top four best-scoring ligands of these twenty compounds are shown in Table 3; the remaining ligands are shown here. Listed with each compound is the AutoDock-predicted binding energy of the most-populated AutoDock cluster (Energy), as well as chemical properties computed using ICM 3.7, including molecular weight (Weight), the number of hydrogen bond acceptors (HBA), the number of hydrogen bond donors (HBD), and the predicted LogP.
